# Targeting the JAK-STAT pathway in colorectal cancer: mechanisms, clinical implications, and therapeutic potential

**DOI:** 10.3389/fcell.2024.1507621

**Published:** 2024-11-26

**Authors:** Penghui Li, Di Huang

**Affiliations:** ^1^ Department of Gastrointestinal Surgery, The First Affiliated Hospital, College of Clinical Medicine, Henan University of Science and Technology, Luoyang, Henan, China; ^2^ Department of Child Health Care, The Third Affiliated Hospital of Zhengzhou University, Zhengzhou, Henan, China

**Keywords:** JAK-STAT, colorectal cancer, mechanism, prognosis, therapeutic potential

## Abstract

Colorectal cancer (CRC) remains one of the most prevalent and fatal malignancies worldwide, consistently ranking among the top three in terms of incidence and mortality. Despite notable advancements in early detection and therapeutic interventions, survival outcomes for advanced-stage CRC are still dismal, largely due to issues such as drug resistance and metastasis. Recent research has increasingly implicated the JAK-STAT signaling pathway as a pivotal contributor to CRC pathogenesis. This evolutionarily conserved pathway plays a key role in transmitting extracellular signals to the nucleus, thereby modulating gene expression involved in numerous fundamental biological processes. In CRC, dysregulation of the JAK-STAT pathway is frequently observed and is strongly associated with tumor progression, including processes such as cellular proliferation, apoptosis, metastasis, immune evasion, and the sustenance of cancer stem cells. Given its integral role in CRC advancement, the JAK-STAT pathway has gained recognition as a viable therapeutic target. Extensive evidence from preclinical and clinical models supports the efficacy and safety of targeting components of the JAK-STAT pathway, presenting new therapeutic possibilities for patients with CRC, particularly in addressing drug resistance and enhancing treatment outcomes. This review offers a detailed exploration of the JAK-STAT pathway, focusing on its regulatory mechanisms in CRC-related malignancies. Moreover, it examines the association between JAK-STAT protein expression, clinical features, prognosis, and its therapeutic potential in CRC management.

## 1 Introduction

Colorectal cancer (CRC) remains a highly prevalent and lethal malignancy globally (Midgley and Kerr, 1999; Siegel et al., 2020; Li N. et al., 2021; Ranasinghe et al., 2022). According to the latest Global Cancer Statistics 2022, CRC ranks third in incidence and second in cancer-related mortality in the United States (Rim et al., 2009; Biller and Schrag, 2021; Bray et al., 2024). In 2024, approximately 152,810 new cases were projected to be diagnosed, with an estimated 53,010 deaths resulting from the disease. Current CRC treatment approaches involve a combination of surgery, chemotherapy, radiotherapy, and targeted therapies, tailored to the tumor’s stage and molecular profile (Mármol et al., 2017; Wong and Yu, 2019; Liu Y. et al., 2023). For advanced and metastatic CRC, systemic chemotherapy regimens such as FOLFOX (5-fluorouracil, leucovorin, and oxaliplatin) or FOLFIRI (5-fluorouracil, leucovorin, and irinotecan) are commonly employed (Saltz, 2013; Tabernero et al., 2014; Venook et al., 2017; Stintzing et al., 2019; Guo et al., 2021). Prognosis is heavily influenced by the stage at diagnosis, with a significant decline in survival rates for advanced CRC, where the 5-year survival rate falls below 15% (Pickhardt et al., 2011; Nguyen and Weinberg, 2016; Rumpold et al., 2020; Shin et al., 2023). Despite advancements in screening and therapeutic strategies, the emergence of drug resistance and the limited availability of effective treatment options remain substantial challenges, particularly for patients with advanced disease (Andrei et al., 2022; Xing et al., 2023; Cañellas-Socias et al., 2024). Overcoming therapeutic resistance and developing novel treatment modalities for advanced and metastatic CRC are crucial for improving patient outcomes (Van Cutsem et al., 2013; Fessler and Medema, 2016; Zhang et al., 2023).

The Janus kinase (JAK)-signal transducer and activator of transcription (STAT) pathway plays a critical role in transmitting signals from extracellular cytokines and growth factors to the nucleus, modulating gene expression that governs cell proliferation, angiogenesis, immune responses, and survival (Hu et al., 2021; Chan et al., 2022; Hu et al., 2023; Xue et al., 2023). Beyond its role in maintaining normal physiological processes, the JAK-STAT pathway contributes to the pathogenesis of various disorders, including autoimmune diseases, hematologic malignancies, and solid tumors (O'Shea et al., 2015; Trivedi and Starz-Gaiano, 2018; Gutiérrez-Hoya and Soto-Cruz, 2020; Erdogan et al., 2022). In CRC, dysregulation of the JAK-STAT pathway has been widely reported, with abnormal signaling profoundly affecting critical biological processes, such as cellular proliferation, survival, migration, invasion, cancer stem cell maintenance, and immune evasion (Liang et al., 2019; Zheng et al., 2022; Ghasemian et al., 2023). Given its central role in CRC progression, targeting the JAK-STAT pathway has emerged as a promising therapeutic approach (Silva et al., 2021; Yang et al., 2022; Lu et al., 2023). Numerous studies have demonstrated the effectiveness and safety of JAK-STAT inhibitors in CRC models, showing reduced tumor growth, increased therapeutic sensitivity, and enhanced immune responses (Chalikonda et al., 2021; Bajpai et al., 2024; Wu et al., 2024).

This review offers an in-depth examination of the JAK-STAT pathway, with a focus on its regulatory mechanisms in CRC-related malignancies. Additionally, we discuss the clinical relevance of JAK-STAT protein expression, its prognostic value, and the potential of targeting this pathway as a therapeutic strategy in CRC management.

## 2 Overview of the JAK-STAT pathway

### 2.1 The JAK-STAT pathway composition

The JAK-STAT signaling pathway represents a highly efficient mechanism for transmitting extracellular signals directly to the nucleus (Vainchenker and Constantinescu, 2013; Xin et al., 2020; Park et al., 2023; Liang et al., 2024). The core components of this pathway include ligands, receptors, JAKs, STAT proteins (Signal Transducers and Activators of Transcription), and regulatory proteins such as Suppressors of Cytokine Signaling (SOCS).

In humans, JAKs belong to the non-receptor tyrosine kinase family and are composed of four members: JAK1, JAK2, JAK3, and TYK2 (Banerjee et al., 2017; Damerau et al., 2020; Pang et al., 2023; Liang et al., 2024). Each of these JAKs associates selectively with specific cytokine and hormone receptors, contributing to their distinct biological functions. JAK3, in particular, is predominantly expressed in the bone marrow, lymphatic system, endothelial cells, and vascular smooth muscle cells, whereas the other JAK members are typically present in most tissues. Structurally, JAK proteins are organized into several domains: the N-terminal FERM domain, which mediates interactions with receptors; the Src homology 2 (SH2)-like domain, which binds phosphorylated tyrosine residues; the pseudokinase domain, which exerts regulatory control; and the C-terminal tyrosine kinase domain, responsible for phosphorylating downstream targets.

The STAT family includes seven proteins: STAT1, STAT2, STAT3, STAT4, STAT5a, STAT5b, and STAT6 (Li, 2008; Yu et al., 2014; Verhoeven et al., 2020). In their inactive state, STAT proteins are localized in the cytoplasm, where they consist of six conserved domains that serve diverse functional roles. These domains include the N-terminal domain, which facilitates STAT dimerization; the coiled-coil domain, which regulates nuclear import and export; the DNA-binding domain, which interacts with gene promoters; the linker domain; the SH2 domain, which recognizes phosphorylated cytokine receptors; and the C-terminal transcriptional activation domain (TAD), which activates the transcription of target genes.

### 2.2 The JAK-STAT pathway activation and regulation

**FIGURE 1:**
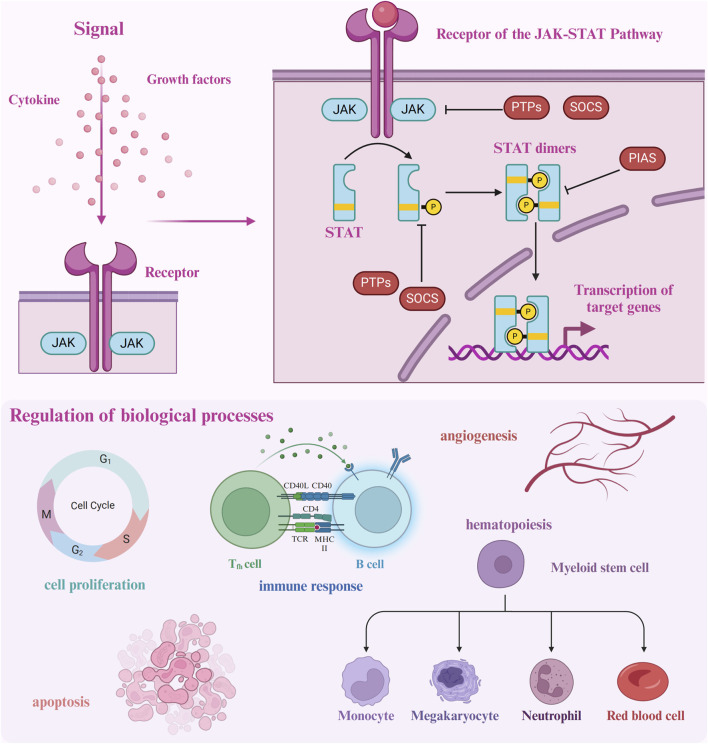
Upon ligand binding to its receptor, the JAK-STAT pathway is initiated by receptor dimerization, which facilitates the proximity of JAK proteins, enabling mutual phosphorylation as well as phosphorylation of the receptor. Phosphorylated tyrosine residues on the receptor serve as docking sites for STAT proteins, which are subsequently phosphorylated by JAKs. The activated STAT proteins dimerize and translocate to the nucleus, where they bind to DNA and regulate gene transcription. The pathway is tightly controlled by several regulatory mechanisms: SOCS proteins inhibit JAK activity, while PIAS and PTPs modulate and dephosphorylate STATs, respectively, ensuring precise signal termination.

The JAK-STAT pathway orchestrates several critical biological functions, including cellular processes, immune regulation, hematopoiesis, angiogenesis, and oncogenesis (Hou et al., 2002; Amoyel and Bach, 2012). This pathway is activated when cytokines or growth factors bind to their respective receptors on the cell surface, inducing conformational changes that lead to the activation of JAK proteins (Müller et al., 2008; La Fortezza et al., 2016). Once activated, JAKs phosphorylate each other and tyrosine residues on the receptor’s cytoplasmic domain, creating docking sites for STAT proteins. The SH2 domains of STATs bind to these phosphorylated residues, positioning them for subsequent phosphorylation by JAKs. Phosphorylated STATs then dimerize and translocate to the nucleus, where they regulate the expression of target genes involved in diverse cellular processes. Cytokine or growth factor binding serves as the primary trigger for the JAK-STAT signaling cascade, ultimately resulting in changes to gene expression (Figure 1).

The activation of the JAK-STAT pathway is tightly regulated through several negative feedback mechanisms to prevent excessive or prolonged signaling (Liu J. et al., 2023; López-Mejía et al., 2023). A primary regulatory mechanism involves the Suppressor of Cytokine Signaling (SOCS) proteins, which include SOCS1-7 and the cytokine-inducible SH2-containing protein (CIS) (Krebs and Hilton, 2001; Yoshimura et al., 2007; Kopalli et al., 2022; Pandey et al., 2023). Once JAK-STAT signaling is initiated, SOCS proteins bind to phosphorylated JAKs or receptors, targeting them for proteasomal degradation and thereby halting further activation of the pathway. Other key regulatory proteins include the protein inhibitors of activated STAT (PIAS) and protein tyrosine phosphatases (PTPs) (Tanuma et al., 2001; Xu and Qu, 2008; Luo et al., 2022; Tobin and Cooper, 2024). PIAS proteins inhibit STATs by either preventing their binding to DNA or promoting their sumoylation, which deactivates their transcriptional activity. PTPs, such as SHP-1 and SHP-2, dephosphorylate JAKs and STATs, reversing their activation and effectively terminating the signaling process (Niu et al., 2018; Taheri et al., 2018; Ghafouri-Fard et al., 2021).

Under normal physiological conditions, the JAK-STAT pathway is indispensable for transmitting signals from extracellular cytokines and growth factors to regulate essential biological functions. However, persistent activation of this pathway has been implicated in the pathogenesis of various diseases, particularly cancer and chronic inflammatory conditions (Banerjee et al., 2017; Xin et al., 2020; Sun et al., 2023).

## 3 Involvement of the JAK-STAT pathway in CRC

**FIGURE 2:**
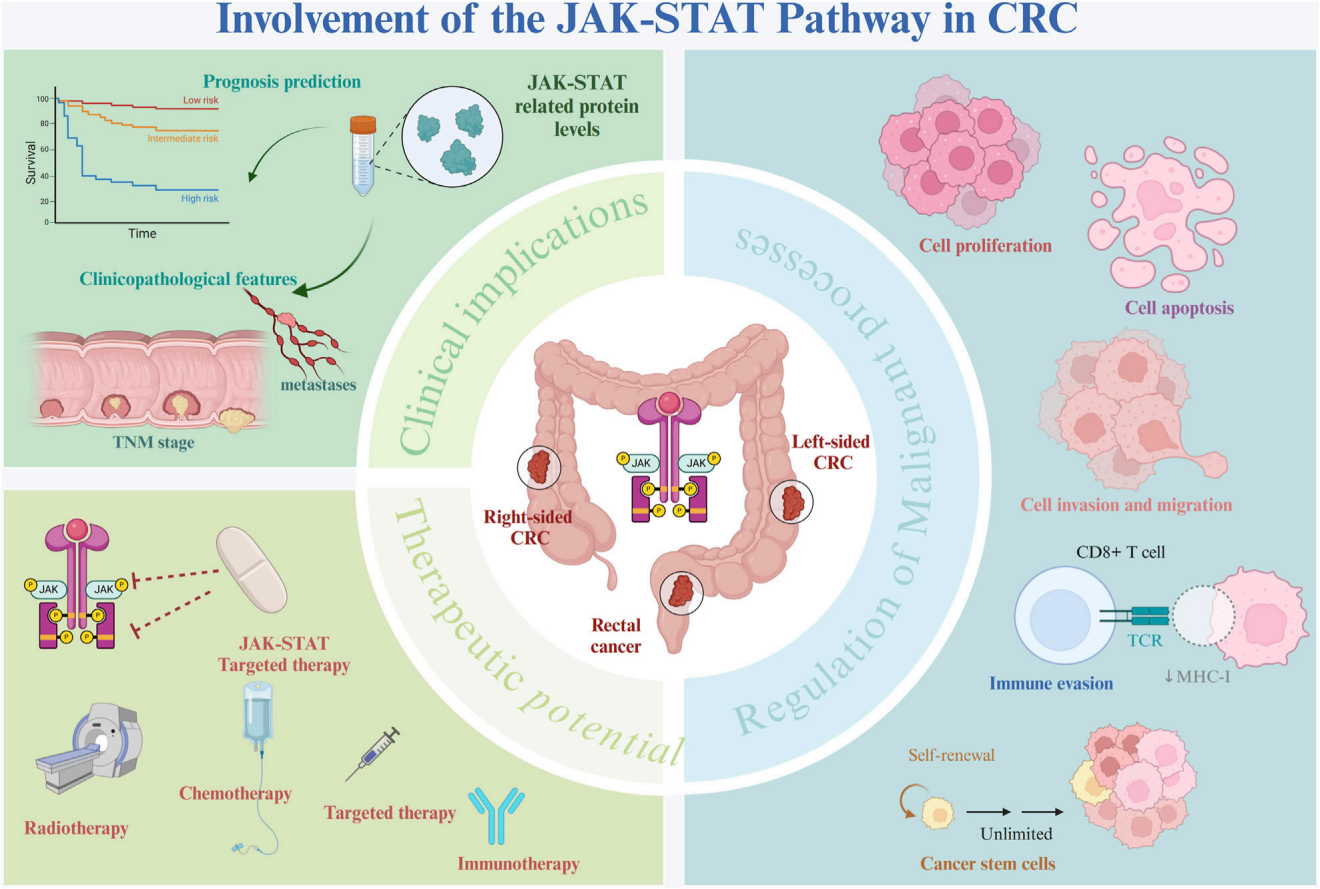
Role of the JAK-STAT pathway in CRC. The JAK-STAT pathway plays a pivotal role in the pathogenesis of CRC. Aberrant activation of the JAK-STAT pathway interacts with several oncogenic molecules and pathways, driving several key biological processes, including cell proliferation, migration, invasion, immune evasion, and the maintenance of cancer stem cell phenotypes. Dysregulation of the JAK-STAT pathway has been strongly linked to patient prognosis and various adverse clinical characteristics, making it a significant biomarker of CRC progression. Targeting the JAK-STAT pathway represents a promising therapeutic strategy that could potentially improve CRC treatment outcomes.

Abnormalities in the JAK-STAT pathway have been documented across various malignancies, including breast, colorectal, esophageal, liver, lung, ovarian, pancreatic, and prostate cancers (Xue et al., 2021; Fernández et al., 2023; Mahjoor et al., 2023; Jia et al., 2024). In recent years, growing evidence has underscored the pivotal role this pathway plays in CRC development. Dysregulated JAK-STAT signaling contributes to CRC progression by modulating essential processes such as cell proliferation, apoptosis, migration, invasion, stem cell phenotype maintenance, and immune evasion (Figure 2). Given the extensive involvement of this pathway in malignant behaviors, this section will focus on elucidating the molecular mechanisms and biological functions of the JAK-STAT pathway in key tumorigenic processes in CRC (Table 1).

**TABLE 1:** Molecular mechanisms of the dysregulated JAK-STAT pathway in CRC.

Upstream	JAK-STAT components	Downstream	Molecular mechanisms	Biological processes	Cell lines	Publication year	Ref.
MS4A4A	JAK/STAT6	—	MS4A4A/JAK/STAT6	Promote M2 macrophage polarization, T-cell exhaustion, and inhibit effector CD8^+^ T cell infiltration	Human CRC cell lines (SW480, HT29, LoVo and RKO), mouse CRC cell lines (MC38, CT26), human monocyte macrophage cell line (THP-1), and mouse macrophage cell line (J774A.1, RAW264.7)	2023	Li Y. et al. (2023)
Lactate/METTL3/YTHDF1	JAK1/STAT3	—	Lactate/METTL3/YTHDF1/JAK1 /STAT3	Promote immune escape	mouse CRC cell line (MC38)	2022	Xiong et al. (2022)
—	JAK1/JAK2/STAT3	—	—	Promote cell viability and proliferation	human CRC cell lines (DLD-1, HT29, SW620, HCT116, SW837 and CCD18Co)	2024	Pennel et al. (2024)
—	JAK2/STAT3	CCND2	JAK2/STAT3 /CCND2	Promote cancer stem cell persistence and radioresistance	patient-derived primary CRC cells, human CRC cell lines (LoVo and HCT116)	2019	Park et al. (2019)
circSPARC	JAK2/STAT3	—	circSPARC/JAK2 /STAT3	Promote cell viability, clone formation ability, invasion and proliferation	human CRC cell lines (HCT116, SW620, SW480, DLD1, HT-29, and LoVo)	2021	Wang et al. (2021)
—	JAK1/JAK2/STAT5	LY6G6D	JAK/STAT5 /LY6G6D	Promote cell proliferation and inhibit cell apoptosis	human CRC cell lines (HCT116, SW480, and RKO)	2019	Giordano et al. (2019)
SRC-1/SOCS1	JAK1/STAT3	PD-L1	SRC-1/SOCS1 /JAK1/STAT3 /PD-L1	Reduce tumor infiltration and vitality of CD8^+^ T cells to promote immune escape	murine CRC cell lines (CMT93, CT26, MC38) and human CRC cell lines (HCT116)	2024	Hong et al. (2024)
Vitamin D	JAK2/STAT3	—	Vitamin D/VDR /JAK2/STAT3	Promote dysbiosis and shift bacterial profile from normal to susceptible carcinogenesis	Human CRC cell lines (HCT116)	2020	Zhang et al. (2020)
Triptolide/Rac1 /IL6R	JAK1/STAT3	—	Triptolide/Rac1 /IL6R/JAK1 /STAT3	Promote cell proliferation and survival	Human CRC cell lines (Caco2 and SW480)	2009	Wang et al. (2009)
∆133p53	JAK/STAT3	RhoA/ROCK/NF-κB	∆133p53/JAK /STAT3/RhoA /ROCK/NF-κB	Promote tumor invasion and metastasis	Human CRC cell lines (HCT116)	2018	Campbell et al. (2018)
APC1	JAK/STAT3	—	Apc1/EGFR /Wnt/MYC /JAK/STAT3	Promote intestinal hyperproliferation	—	2012	Cordero et al. (2012)
IFN-γ	JAK/STAT	PD-L1	IFN-γ/JAK /STAT/PD-L1	Promote tumor-shielding responses	Human CRC cell lines (HCT116 and SW480) and murine CRC cell lines (CT26)	2019	Yuan et al. (2019)
MEK	JAK1/STAT1	MHCI	MEK1/JAK1 /STAT1/MHCI	Promote antigen presentation	murine CRC cell lines (CT26)	2021	Dennison et al. (2021)
IL-1β/TGF-β	JAK/STAT3	—	IL-1β/TGF-β /JAK/STAT3	Promote chemoresistance	Human CRC cell lines (DLD-1, HT29, and HCT116) and primary normal colorectal fibroblasts	2019	Guillén Díaz-Maroto et al. (2019)
NSC13626	JAK2/STAT3	—	NSC13626/JAK2 /STAT3	Promote cell proliferation	Human CRC cell lines (HCT116 and HT-29)	2018	Lin et al. (2018)
IL-11	JAK/STAT3	IFN-γ and TNF-α	IL-11/JAK /STAT3/IFN-γ and TNF-α	Suppress CD4^+^ T cell-mediated antitumor responses	murine CRC cell lines (CT26)	2021	Huynh et al. (2021)
Genistein	STAT3	—	Genistein/STAT3	Promote cell proliferation	Human CRC cell lines (HCT 116 and HT-29)	2020	Dariya et al. (2021)
LIF/BRD4	STAT3	—	IKK-α/NF-κB /LIF/BRD4 /JAK/STAT3	Inhibit cell apoptosis, and promote DNA damage repair and chemoresistance	Human CRC cell lines (HCT116, Caco2, and HT29)	2023	Pecharromán et al. (2023)
CMTR1	STAT3	—	CMTR1/STAT3	Promote immune evasion and cell growth	Human CRC cell lines (HCT116, SW480, and RKO)	2023	You et al. (2023)
lncRNA RP11-468E2.5	STAT5 and STAT6	—	lncRNA RP11-468E2.5 /STAT5 and STAT6	Promote cell proliferation and inhibit cell apoptosis	Human CRC cell lines (RKO, LOVO, SW620, SW480, and HCT116)	2019	Jiang et al. (2019)
NF-kB/p65/Progranulin	STAT3	—	NF-kB/p65 /Progranulin/STAT3	Promote cell proliferation and survival	Human CRC cell lines (HCT116 and HT-29)	2019	Laudisi et al. (2019)
AAG8	STAT3	—	AAG8/STAT3	Promote cell proliferation	Human CRC cell lines (DLD-1 and HCT116)	2014	Sun et al. (2014)
—	STAT1/STAT3	CD44	STAT1/STAT3 /CD44	Promote cancer stem cell persistence	Human CRC cell lines (HCT116) and murine CRC cell lines (CT26 and MC38)	2023	Jing et al. (2023)
LN521	STAT3	CD44	LN521/STAT3 /CD44	Promote cell invasion and self-renewal	Human CRC cell lines (DLD-1) and patient-derived CRC liver metastasis cells (CPP19)	2020	Qin et al. (2020)
—	STAT3	—	—	Promote cell proliferation, invasion, and migration	Human CRC cell lines (HCT116)	2024	Fan et al. (2024)
—	STAT3	—	—	Promote stemness properties	Human CRC cell lines (HCT116 and HT29)	2018	Chung et al. (2018)
—	STAT3	—	—	—	—	2018	Jonker et al. (2018)
—	STAT3	—	—	—	—	2021	Taniguchi et al. (2021)
—	STAT3	—	—	—	—	2023	Shah et al. (2023)
—	Unphosphorylated STAT5A	HP1α	Unphosphorylated STAT5A/HP1α	Promote heterochromatin formation and suppress tumor growth	Human CRC cell lines (DLD-1)	2013	Hu et al. (2013)
—	JAK/STAT /SOCS	—	—	—	—	2013	Slattery et al. (2013)
—	SOCS3	CD163	SOCS3/CD163	Correlate with lung metastasis and immune cell infiltration	—	2023	Li X. et al. (2023)

### 3.1 Cell proliferation, apoptosis, migration, and invasion

In CRC, upregulated circSPARC functions as a miR-485-3p sponge, which leads to increased JAK2 expression and the subsequent phosphorylation of STAT3. Moreover, circSPARC binds to FUS, promoting the nuclear translocation of phosphorylated STAT3 (p-STAT3). Activation of the JAK2-STAT3 signaling pathway enhances the viability, clonogenic potential, invasion, and proliferation of CRC cells, including HCT116 and DLD1, thus accelerating CRC progression (Wang et al., 2021). The IκB kinase (IKK) complex has also been frequently implicated in CRC initiation and advancement (Liu et al., 2020; Patel et al., 2022; Yu et al., 2022). Recent research has identified a functional interaction between IKK-α, BET family protein BRD4, and the JAK-STAT pathway. IKK-α phosphorylates BRD4 at serine 1,117, strengthening its interaction with STAT3 and activating the DNA damage response kinases ATM and Chk1 (Pecharromán et al., 2023). This process is mediated by NF-κB-driven induction of leukemia inhibitory factor (LIF), leading to enhanced STAT3 activation. As a result, BRD4-STAT3 complexes form, triggering the JAK-STAT3 pathway, which inhibits apoptosis and promotes DNA damage repair in human CRC cells (Pecharromán et al., 2023).

Additionally, increasing evidence has demonstrated that Vitamin D confers a protective effect against CRC, acting through a vitamin D receptor (VDR)-dependent mechanism (Pálmer et al., 2003; Fernandez-Garcia et al., 2005; Chan and Giovannucci, 2010).

Notably, reduced vitamin D receptor (VDR) levels, in conjunction with increased bacterial colonization and elevated JAK2 and STAT3 expression, have been identified in both human CRC samples and azoxymethane/dextran sulfate sodium (AOM/DSS)-induced CRC models (Zhang et al., 2020). VDR deficiency in the gut contributes to the hyperactivation of the JAK2-STAT3 signaling pathway, leading to gut microbiota dysbiosis and heightened susceptibility to carcinogenesis (Zhang et al., 2020). Furthermore, nuclear factor κB (NF-κB)/p65 activation perpetuates the expression of the STAT3 cofactor progranulin in human CRC cells, which in turn augments STAT3 activation, promoting CRC cell proliferation and survival (Laudisi et al., 2019). The TP53 gene, which encodes 12 isoforms, exhibits notable dysregulation of the ∆133p53 isoform in human tumors, contributing to tumorigenesis (Fujita et al., 2009; Bernard et al., 2013; Gadea et al., 2016; Joruiz et al., 2020). The oncogenic function of ∆133p53 is reliant on Interleukin-6 (IL-6), which constitutively activates the JAK-STAT3 pathway, subsequently triggering GTPase RhoA and its associated kinase ROCK. This cascade activates NF-κB, promoting the secretion of pro-inflammatory chemokines and fostering an invasive CRC phenotype (Campbell et al., 2018). Triptolide, a diterpenoid triepoxide derived from traditional Chinese medicinal herbs, has demonstrated efficacy in disrupting the IL6R-JAK1-STAT3 pathway by inactivating small GTPase Rac1, thereby reducing CRC incidence, improving survival rates in colitis-associated CRC mouse models, and inhibiting proliferation in CRC cell lines such as SW480 and Caco-2 (Wang et al., 2009).

**FIGURE 3:**
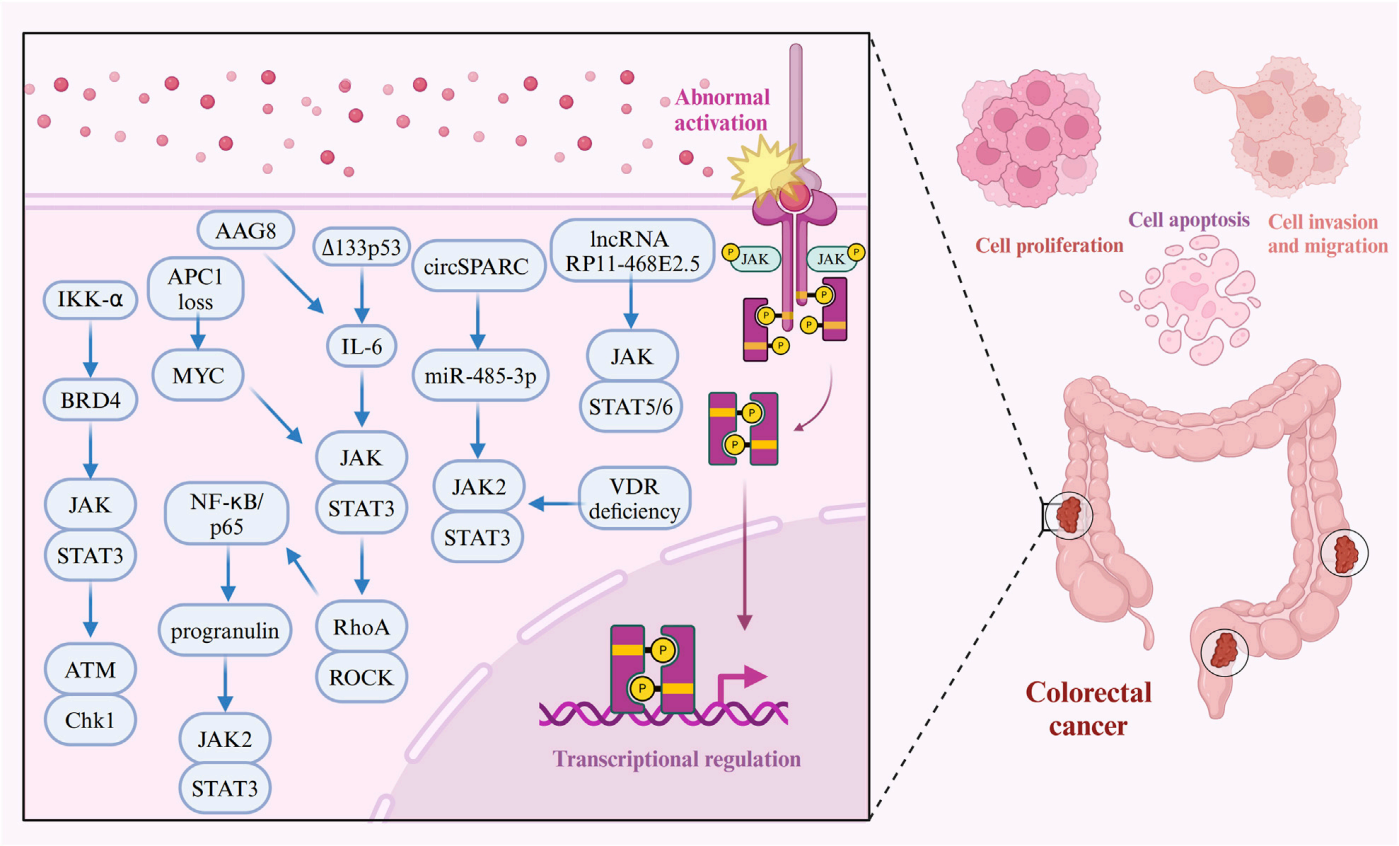
Molecular mechanisms of the JAK-STAT pathway in CRC cell proliferation, apoptosis, migration, and invasion. The JAK-STAT signaling pathway plays a pivotal role in promoting CRC cell proliferation, apoptosis resistance, migration, and invasion through interactions with various oncogenic molecules. For instance, circSPARC functions as a miR-485-3p sponge, leading to the upregulation of JAK2 and subsequent phosphorylation of STAT3, which enhances cell viability and invasiveness. BRD4, *via* IKK-α phosphorylation, interacts with STAT3 to facilitate DNA damage repair and tumor progression. Inflammatory cytokines such as IL-6 and NF-κB further activate STAT3, driving cancer cell survival and metastasis. Additionally, APC mutations fuel JAK-STAT signaling through the Wnt/MYC pathway, while VDR deficiency and gut microbiota dysbiosis amplify JAK2-STAT3 hyperactivation, further contributing to CRC development. Furthermore, molecules like progranulin, ∆133p53, and RhoA reinforce STAT3 signaling, driving CRC progression and promoting resistance to apoptosis.

Aging-associated gene 8 protein (AAG8), a chaperone protein involved in endoplasmic reticulum (ER)-associated degradation, has emerged as an oncogenic factor in CRC. AAG8 promotes cancer cell proliferation by activating STAT3 independently of the IL6-JAK pathway (Sun et al., 2014). Additionally, mutations that inactivate the adenomatous polyposis coli (APC) gene, a key negative regulator of Wnt signaling, are major drivers of both sporadic and hereditary CRC (Ireland et al., 2004; Andreu et al., 2005; Hsu et al., 2007). The loss of APC triggers MYC upregulation and JAK-STAT3 pathway activation, fueling excessive intestinal stem cell (ISC) proliferation in the *Drosophila* midgut in response to heightened Wnt signaling (Cordero et al., 2012). Epidermal growth factor receptor (EGFR) signaling further establishes a positive feedback loop, linking Wnt/MYC activation with JAK-STAT3 pathway signaling in response to APC1 loss, amplifying this proliferative response (Cordero et al., 2012). The interplay between tumor cells and cancer-associated fibroblasts (CAFs) is also pivotal in driving cancer progression and metastasis (Hu et al., 2019; Gong et al., 2020; Kobayashi et al., 2022; Koncina et al., 2023). IL-1β and TGF-β1 are key factors that recruit fibroblasts and transform them into CAFs in CRC. Once activated, CAFs secrete pro-inflammatory mediators that activate the JAK-STAT3 and PI3KCA-AKT pathways, accelerating cancer progression (Guillén Díaz-Maroto et al., 2019). Furthermore, STAT5 and STAT6 are highly expressed in various CRC cell lines and tissues. The long non-coding RNA (lncRNA) RP11-468E2.5 interacts with STAT5 and STAT6, and its upregulation reduces phosphorylation levels of these proteins, leading to inhibited proliferation and the induction of G1/G0 phase arrest and apoptosis in CRC cells (Jiang et al., 2019). In contrast, unphosphorylated STAT5A functions as a tumor suppressor by binding to heterochromatin protein 1α (HP1α), stabilizing heterochromatin, and repressing oncogene expression, thereby inhibiting tumor growth in mouse CRC xenograft models (Figure 3) (Hu et al., 2013).

### 3.2 Immune evasion

In CRC, the tumor microenvironment (TME) plays a pivotal role in promoting immune evasion (Chandra et al., 2021; Che et al., 2021; Kasprzak, 2021). One prominent factor is membrane-spanning four domains subfamily A member 4A (MS4A4A), which is significantly overexpressed in tumor-associated macrophages (TAMs). This overexpression activates JAK-STAT6 signaling, promoting M2 macrophage polarization and CD8^+^ T-cell dysfunction, thereby fostering an immunosuppressive TME that enables the tumor to escape immune surveillance (Li Y. et al., 2023). Metabolic alterations within the TME further exacerbate immune evasion. For example, lactate accumulation in the CRC TME has been shown to upregulate methyltransferase-like 3 (METTL3) in tumor-infiltrating myeloid cells (TIMs) (Li et al., 2024). In conjunction with YTH N6-methyladenosine RNA Binding Protein 1 (YTHDF1), METTL3 mediates m6A modification on JAK1 mRNA, enhancing JAK1 and STAT3 expression and promoting tumor immune escape (Xiong et al., 2022). Another mechanism involves cap methyltransferase 1 (CMTR1), a recently identified oncogene in CRC. CMTR1 binds strongly to the transcription start site (TSS) of STAT3, driving its expression and activation. Activated STAT3, in turn, promotes CMTR1-driven cell growth and immune evasion by suppressing the expression of chemokines and pro-inflammatory factors, thus further advancing tumor progression (You et al., 2023). Additionally, the transcription coactivator steroid receptor coactivator-1 (SRC-1) plays a pivotal role in regulating immune responses in CRC. SRC-1 suppresses SOCS1 expression, leading to the activation of JAK1-STAT3 signaling, which upregulates PD-L1 expression and reduces CD8^+^ T-cell infiltration, thereby diminishing antitumor immunity (Hong et al., 2024). Notably, CRC tumors with high microsatellite instability (MSI-H) exhibit increased responsiveness to PD-1 blockade therapy (Kim et al., 2016; Gong et al., 2017; Lee et al., 2017; Korehisa et al., 2018). Research suggests that low doses of interferon-gamma (IFN-γ) significantly elevate PD-L1 expression in MSI-H cell lines, such as HCT116, likely *via* the JAK-STAT pathway. Specifically, PD-L1 expression in these cells is primarily driven by the JAK3-STAT5A/5B pathway. Overexpression of PD-L1 not only enhances apoptosis resistance in CRC CT26 cells but also limits CD8^+^ T-cell infiltration, contributing to immune evasion and facilitating tumor growth (Yuan et al., 2019). Furthermore, interleukin-11 (IL-11) has emerged as a key player in the immunomodulatory landscape of CRC. By activating the JAK-STAT3 pathway, IL-11 inhibits the production of critical pro-inflammatory cytokines such as IFN-γ and TNF-α by tumor-infiltrating CD4^+^ T cells. This inhibition supports the creation of an immunosuppressive environment, which aids in CRC cell proliferation and survival (Huynh et al., 2021).

### 3.3 Stem cell characteristics

Cancer stem cells (CSCs) play a pivotal role in tumor growth, metastasis, drug resistance, and recurrence (Frank et al., 2021; Zheng et al., 2022; Zhu et al., 2022; Zhao et al., 2024). Napabucasin (BBI608), an orally administered STAT3 inhibitor, has undergone clinical evaluation for treating various cancers (Hubbard and Grothey, 2017; Froeling et al., 2019; Bitsch et al., 2022; Bekaii-Saab et al., 2023; Jing et al., 2023). *In vitro* studies have revealed that Napabucasin exerts potent tumor-suppressive effects by specifically targeting CSCs in CRC. It achieves this by inhibiting STAT3 and STAT1 signaling, leading to a reduction in CD44 expression, a key marker linked to CSCs (Jing et al., 2023). Additionally, laminin 521 (LN521) has been shown to promote the self-renewal and invasion of CRC cells by enhancing STAT3 phosphorylation, an effect that can be counteracted by Napabucasin (Qin et al., 2020). In radioresistant CRC tissues, the JAK/STAT signaling pathway is hyperactivated, which is associated with both local and distant metastasis. Research indicates that CRC stem cell subpopulations exposed to radiotherapy (RT) tend to overexpress JAK2, resulting in increased STAT3 phosphorylation. This heightened activation drives the transcription of cyclin D2 (CCND2), promoting CSC self-renewal and contributing to the persistence of radioresistance, further complicating treatment outcomes (Park et al., 2019).

## 4 Clinical implications and treatment potentials of the JAK-STAT pathway in CRC

### 4.1 Clinical implications of the JAK-STAT pathway in CRC

Emerging research increasingly links abnormal JAK-STAT signaling to CRC prognosis and clinicopathological characteristics, including tumor invasion depth, size, and metastasis (Table 2) (Amoyel et al., 2014; Jia et al., 2024). Multiple studies have demonstrated a significant association between elevated JAK-STAT3 expression in various CRC cell lines and poorer survival in patients with high stromal invasion (TSPhigh). This correlation has been observed across the TransSCOT study and two large retrospective CRC cohorts (Glasgow combined array and Glasgow Royal Infirmary array) (Pennel et al., 2024). Additionally, population-based case-control studies have highlighted the importance of the JAK-STAT-SOCS signaling pathway in CRC development, where genetic variants in this pathway are linked to an increased risk of both colon and rectal cancers. Furthermore, several single nucleotide polymorphisms (SNPs) in JAK-STAT pathway genes have been associated with survival outcomes in patients with CRC, underscoring their potential as prognostic markers (Slattery et al., 2013). Whole exome sequencing of tissues from metachronous CRC liver metastases (mCLM) and matched non-malignant liver samples has revealed that genetic alterations within the JAK-STAT pathway may serve as indicators of longer overall survival (OS) in patients with mCLM (Heczko et al., 2023). Additionally, phosphorylated STAT5 (p-STAT5) and phosphorylated STAT6 (p-STAT6) are expressed at significantly higher levels in CRC tissues compared to adjacent normal tissues. Higher expression levels of these phosphorylated proteins correlate with deeper tumor invasion, suggesting a more aggressive tumor phenotype (Jiang et al., 2019). Recent findings have also identified lymphocyte antigen 6 complex locus G6D (LY6G6D) as a carcinogenic antigen in microsatellite stable (MSS) CRC. In an analysis of CRC subtypes stratified by mismatch repair (MMR) status (TCGA, n = 276), STAT5 and LY6G6D were more highly expressed in MSS tumors compared to microsatellite instability (MSI) tumors. Metastatic cell lines, such as SW620, characterized by elevated LY6G6D expression, also demonstrated the highest levels of p-STAT5. Importantly, positive staining of p-STAT5 and LY6G6D in normal mucosa and CRC samples was directly linked to shorter survival times for patients with CRC (Giordano et al., 2019). Further analysis of The Cancer Genome Atlas (TCGA) data revealed a strong positive correlation between mRNA expression levels of STAT1, STAT2, and STAT3 with CD44, suggesting that JAK-STAT signaling is closely associated with the maintenance of stemness in CRC stem cells (Jing et al., 2023). Additionally, both TCGA data and immunohistochemical (IHC) analysis of 32 CRC primary tumors, metastases, and normal intestinal mucosa from patients with lung metastases have suggested a key role for SOCS3 in CRC progression. SOCS3 expression is closely linked to macrophage infiltration and CRC lung metastasis, with patients presenting lymph node and distant metastases at diagnosis often showing elevated SOCS3 expression in lung metastasis sites (Li X. et al., 2023). Lung metastases also exhibit higher levels of both CD163 and SOCS3 compared to primary tumors, indicating SOCS3’s potential role in promoting CRC metastasis and influencing patient prognosis (Li X. et al., 2023).

**TABLE 2:** Clinical correlation of the JAK-STAT pathway and its therapeutic potential in CRC.

JAK-STAT pathway components	Expression/Mutations	Role	Clinical samples and animal models	Clinical significance	Publication year	Ref.
JAK1/JAK2/STAT3	Upregulated	Oncogene	3 independent CRC resected patient cohorts, including the TransSCOT clinical trial cohort, Glasgow combined array, Glasgow royal infirmary array, and mouse organoids, patient-derived organoids and explants	Poor prognosis and therapeutic strategies (JAK1/2 inhibitors)	2024	Pennel et al. (2024)
JAK2/STAT3	Upregulated	Oncogene	the GSE15781, GSE70574, GSE68468 CRC datasets, and	Clinicopathological features (local and distant metastases), and therapeutic strategies (the combination of JAK2 inhibition with RT)	2019	Park et al. (2019)
STAT5 and STAT6	Upregulated	Oncogene	CRC and adjacent normal tissues from 169 patients with CRC, the GSE4107 and GSE21510 datasets, and CRC xenograft models	Clinicopathological features (tumor infiltration depth)	2019	Jiang et al. (2019)
JAK/STAT/SOCS pathway	Genetic Variation	Oncogene	a population-based case-control study of colon cancer (cases n = 1,555; controls n = 1,956) and rectal cancer (cases n = 754; controls n = 959)	Poor prognosis	2013	Slattery et al. (2013)
JAK1/JAK2/STAT5	Upregulated	Oncogene	the TCGA, GSE20916, and GSE41258 datasets, 2 independent datasets of patients with sporadic CRC, fresh tissue specimens from CRC tumor and matched normal adjacent mucosa, tissue microarrays included tumor tissue from 516 CRC and 92 corresponding normal mucosa specimens	Poor prognosis and therapeutic strategies (trametinib and JAK2/JAK1 inhibitor momelotinib)	2019	Giordano et al. (2019)
SOCS3	Upregulated	Oncogene	32 CRC primary tumors and metastases and normal intestinal mucosal samples from patients with lung metastasis	Poor prognosis and correlate immune cell infiltration and lung metastasis	2023	Li X. et al. (2023)
STAT1/STAT3	Upregulated	Oncogene	CRC tumor-bearing mouse models	Diagnose and therapeutic strategies (N3-TMPs@NAP)	2023	Jing et al. (2023)
JAK/STAT6	Upregulated	Oncogene	the TCGA and GSE17538 CRC datasets, and murine subcutaneous tumor and orthotopic transplanted models	Therapeutic strategies (AS1517499, siMs4a4a, anti-MS4A4A monoclonal antibody, and ICIs)	2023	Li Y. et al. (2023)
JAK1/STAT3	Upregulated	Oncogene	human tumor and adjacent normal tissue samples, the subcutaneous tumor model, and tumor colonization model	Therapeutic strategies (METTL3 inhibitor)	2022	Xiong et al. (2022)
STAT3	Upregulated	Oncogene	—	Therapeutic strategies (Genistein)	2020	Dariya et al. (2021)
STAT3	Upregulated	Oncogene	patient-derived and mouse intestinal organoids	Therapeutic strategies (Combined JAK/STAT, IKK-α inhibition, 5-FU and irinotecan)	2023	Pecharromán et al. (2023)
STAT3	Upregulated	Oncogene	the TCGA-COAD cohort, a tissue microarray containing 73 paired human CRC tissues (Cat No. COC1601), and murine subcutaneous tumor and orthotopic transplanted models	Therapeutic strategies (PD1 blockade immunotherapy)	2023	You et al. (2023)
JAK1/STAT3	Upregulated	Oncogene	36 human CRC primary tumor specimens and matched adjacent normal tissues, tissue microarray containing 75 paired human CRC tumors and matched adjacent non-tumor tissues, and CRC xenograft models	Therapeutic strategies (targeting SRC-1 in combination with PD-L1 antibody immunotherapy)	2024	Hong et al. (2024)
STAT3	Upregulated	Oncogene	26 CRC tumor specimens and matched adjacent normal tissues, and human CRC explants	Therapeutic strategies (Progranulin knockdown with chemotherapeutic drugs)	2019	Laudisi et al. (2019)
JAK1/STAT3	Upregulated	Oncogene	a colitis-induced colon cancer mouse model	Therapeutic strategies (Triptolide)	2009	Wang et al. (2009)
STAT3	Upregulated	Oncogene	CRC xenograft models	Therapeutic strategies (combined inhibition of AAG8 and IL6/JAK signalling)	2014	Sun et al. (2014)
—	—	—	396 patients with relapsed/refractory metastatic CRC	Therapeutic strategies (ruxolitinib plus regorafenib)	2018	Fogelman et al. (2018)
JAK/STAT	Upregulated	Oncogene	CRC xenograft models	Therapeutic strategies (PD-1 therapy plus IFN-γ)	2019	Yuan et al. (2019)
JAK1/STAT1	Upregulated	—	56 human blood samples and CRC xenograft models	Therapeutic strategies (the combination of cobimetinib, anti-PD-1, and anti-4-1BB)	2021	Dennison et al. (2021)
JAK/STAT3	Upregulated	—	a patient-derived orthotopic CRC xenograft model	Therapeutic strategies (the combination of a TAK1 inhibitor plus TGFBR1 inhibitor)	2019	Guillén Díaz-Maroto et al. (2019)
JAK2/STAT3	Upregulated	Oncogene	—	Therapeutic strategies	2018	Lin et al. (2018)
unphosphorylated STAT5A	Downregulated	Tumor Suppressor	CRC xenograft models	—	2013	Hu et al. (2013)
JAK/STAT3	Upregulated	Oncogene	the induced sporadic CRC models and CRC xenograft models	—	2021	Huynh et al. (2021)
STAT3	Upregulated	Oncogene	10 liver metastases tissue sections from CRC patients and mouse liver metastasis xenografts	Therapeutic strategies (Napabucasin)	2020	Qin et al. (2020)
STAT3	Upregulated	Oncogene	CRC xenograft models	Therapeutic strategies (SZ6)	2024	Fan et al. (2024)
STAT3	Upregulated	Oncogene	—	Therapeutic strategies (SC-43 and SC-78)	2018	Chung et al. (2018)
STAT3	Upregulated	Oncogene	282 patients with advanced CRC	Therapeutic strategies (Napabucasin with best supportive care)	2018	Jonker et al. (2018)
STAT3	Upregulated	Oncogene	4 Japanese patients with metastatic CRC	Therapeutic strategies (Napabucasin plus FOLFIRI with bevacizumab)	2021	Taniguchi et al. (2021)
STAT3	Upregulated	Oncogene	1,255 patients with previously treated metastatic CRC	Therapeutic strategies (Napabucasin plus FOLFIRI)	2023	Shah et al. (2023)
JAK2/STAT3	Upregulated	Oncogene	84 pairs of CRC tumor tissues and corresponding adjacent normal tissues, 40 preoperative and postoperative CRC plasma samples, and CRC xenografts	—	2021	Wang et al. (2021)
JAK2/STAT3	Upregulated	Oncogene	10 CRC patients with neoplasia and 10 patients without neoplasia, AOM/DSS-induced CRC model,and stem cell-derived colonoids	—	2020	Zhang et al. (2020)
JAK/STAT3	Upregulated	Oncogene	35 CRC tumor specimens, and Δ122p53 IL-6 null mice	—	2018	Campbell et al. (2018)
JAK/STAT3	Upregulated	Oncogene	colon carcinoma tissue arrays, Drosophil, and mouse ISCs	—	2012	Cordero et al. (2012)

### 4.2 Targeting the JAK-STAT pathway as a therapeutic strategy in CRC

A range of agents targeting the dysregulated JAK-STAT pathway has gained notable attention as therapeutic options for precision treatment in CRC. Inhibiting JAK-STAT3 signaling has emerged as a promising strategy for patients with CRC. JAK1/2 inhibitors have demonstrated significant efficacy in reducing cell viability and tumor growth across multiple CRC models, including cell lines, mouse models, and patient-derived organoids (PDOs) (Seyfinejad and Jouyban, 2022; Pennel et al., 2024). However, despite these encouraging preclinical outcomes, a 2018 multicenter, randomized, double-blind phase II clinical trial involving 396 patients revealed that combining the potent JAK1/2 inhibitor Ruxolitinib with regorafenib did not improve OS or progression-free survival (PFS) in patients with relapsed or refractory metastatic CRC when compared to regorafenib plus placebo (Fogelman et al., 2018). Additionally, NSC13626, discovered *via* structure-based virtual screening of the National Cancer Institute (NCI) database, has shown potential as a JAK2 inhibitor. Cellular studies indicate that NSC13626 targets JAK2, leading to reduced STAT3 phosphorylation, arrest of the CRC cell cycle, and suppression of cell growth (Lin et al., 2018). Tumor-derived microparticles (TMPs) from apoptotic tumor cells have also been investigated as potential drug delivery vehicles (Fremder et al., 2014; Phuengkham et al., 2019; Zhao et al., 2019; Akhtar et al., 2023; Chen Y. et al., 2024).

Growing preclinical and clinical evidence suggests that Napabucasin (BBI608), a STAT3 inhibitor, shows considerable promise for CRC treatment (Shao et al., 2023). In a Phase III clinical trial (NCT01830621) across 68 centers with 282 patients with advanced CRC, those with pSTAT3-positive tumors demonstrated a notable survival advantage when treated with Napabucasin and best supportive care compared to the placebo group (median OS 5.1 months vs. 3.0 months) (Jonker et al., 2018). Additionally, a Phase I study in Japan involving four patients with advanced CRC treated with Napabucasin in combination with leucovorin, 5-fluorouracil, irinotecan (FOLFIRI), and bevacizumab exhibited a manageable and tolerable safety profile (Taniguchi et al., 2021). However, in the Phase III CanStem303C trial (NCT02753127) with 1,255 patients with metastatic CRC, Napabucasin combined with FOLFIRI, with or without bevacizumab, failed to improve OS in the general population, though it maintained an acceptable safety profile (Shah et al., 2023). Further supporting Napabucasin’s potential, a recent study introduced the nanoplatform N3-TMPs@NAP, which integrates diagnostic and therapeutic capabilities. PET/CT imaging studies showed that N3-TMPs@NAP exhibited strong antitumor effects by inhibiting STAT1 and STAT3 activation, suppressing CRC CSCs, stimulating T-cell-mediated immune responses, and preventing liver metastasis (Jing et al., 2023). Beyond Napabucasin, other STAT3 inhibitors have shown potential in CRC treatment. The naphthoquinothiazole derivative SZ6 has emerged as a potent STAT3 inhibitor, effectively blocking STAT3 phosphorylation and binding directly to STAT3 (Fan et al., 2024). Moreover, two novel SHP-1 agonists, SC-43 and SC-78, have been identified for their ability to inactivate STAT3 and demonstrate significant antitumor activity by targeting CSC properties (Chung et al., 2018). These SHP-1 agonists have proven more effective than regorafenib in reducing active STAT3 levels. When combined with first-line metastatic CRC therapies, such as oxaliplatin or irinotecan, SHP-1 agonists synergistically diminish CSC subpopulations, further enhancing their therapeutic efficacy.

### 4.3 Combination of JAK-STAT-targeting therapies with existing therapies

Chemotherapy remains a cornerstone in CRC treatment, although its efficacy is often hampered by the emergence of drug resistance and severe side effects. Genistein has gained recognition as a potent chemopreventive agent in CRC (Chen et al., 2020; Rendón et al., 2022; Liao et al., 2023), with molecular docking studies showing its effective interaction with STAT proteins, resulting in inhibited cell proliferation and reduced STAT3 expression in CRC cell lines, including HCT116 and HT-29 (Dariya et al., 2021). Furthermore, combining chemotherapy with inhibitors of IKK-α, STAT3, and BRD4 has shown promise in overcoming chemoresistance in CRC. Specifically, inhibiting STAT3 with Ruxolitinib or an anti-LIF antibody, along with the BRD4 inhibitor JQ1 and the BRAF inhibitor AZ628, has been shown to prevent IKK-α (p45) activation, significantly inducing cell death in CRC cells and patient-derived organoids treated with 5-fluorouracil (5-FU) and irinotecan (Pecharromán et al., 2023). Additionally, the combination of BRAF and JAK-STAT inhibitors has enhanced the long-term efficacy of chemotherapy in CRC xenografts, even in tumors with various mutational profiles, including those resistant to first-line treatments (Pecharromán et al., 2023). Furthermore, progranulin knockdown to downregulate STAT3 sensitizes CRC cells to chemotherapeutic agents like 5-FU, oxaliplatin, and CPT-11, significantly increasing CRC cell death (Laudisi et al., 2019).

In parallel, targeted therapies, such as mitogen-activated protein kinase kinase/extracellular regulated protein kinase (MEK/ERK) inhibitors, have garnered attention for their potential to enhance antitumor immunity through complex immunomodulatory effects on tumor cells and tumor-infiltrating lymphocytes (TILs) (Li H. et al., 2021; Nalli et al., 2021; Tian et al., 2023). Trametinib, a specific inhibitor of MEK1 and MEK2, targets key enzymes in the MAPK/ERK pathway, which are essential for cell division and survival. In CRC tumors expressing LY6G6D and CD15, particularly within the MSS CRC subgroup, combining trametinib with the JAK1/2 inhibitor momelotinib has been found to significantly enhance growth inhibition and apoptosis (Giordano et al., 2019). In mouse models with CT26 MEK1 knockout tumors, marked upregulation of the JAK1-STAT1 pathway, increased MHCI expression, and a higher proportion of activated CD8^+^ T cells led to immune-mediated suppression of CRC tumor growth (Dennison et al., 2021). The JAK-STAT pathway has been identified as a key mediator of immunomodulatory functions in these tumors. However, while cobimetinib, another MEK inhibitor, has been shown to enhance antitumor immunity in tumor cells, it simultaneously impairs T-cell activation, a limitation that can be reversed by treatment with the T-cell agonist anti-4-1BB. The combination of cobimetinib, anti-PD-1, and anti-4-1BB has demonstrated synergistic antitumor activity, significantly improving survival rates and offering a promising therapeutic strategy for CRC (Dennison et al., 2021).

In the past decade, immunotherapy has revolutionized the treatment landscape for a subset of patients with CRC, becoming an increasingly vital option in CRC management (Chen and Wang, 2024). The STAT6 inhibitor AS1517499 has shown significant efficacy in inhibiting M2 macrophage polarization in cells overexpressing MS4A4A. Additionally, siRNA targeting the MS4A4A gene (siMS4A4A) and an anti-MS4A4A monoclonal antibody not only slow tumor growth and reshape the TME but also enhance the efficacy of anti-PD-1 therapy (Li Y. et al., 2023). In C57BL/6J mice implanted with MC38 cells, the combination of CMTR1 knockdown with anti-PD-1 treatment has been shown to significantly increase CD8^+^ T-cell infiltration into the TME and suppress tumor growth, along with increased expression of chemokines and pro-inflammatory factors (You et al., 2023). Moreover, genetic deletion or pharmacological inhibition of SRC-1 using the small-molecule inhibitor bufalin, when combined with PD-L1 blockade, effectively suppresses JAK1-STAT3 signaling, thereby boosting immune responses and producing synergistic antitumor effects (Hong et al., 2024). Currently, pembrolizumab and nivolumab are approved for treating adults with mismatch repair-deficient (dMMR) and MSI-H metastatic or progressive CRC (Riley et al., 2018; Modest et al., 2019; Payandeh et al., 2020; Kanani et al., 2021; Chen X. et al., 2024). However, responses to PD-1 inhibitors among patients with MSI-H CRC can vary (Morse et al., 2020; Bai et al., 2021; Zhang et al., 2022). Recent studies suggest that the MSI-H cell line HCT116 is highly responsive to the IFN-γ-JAK3-STAT5A/5B signaling pathway. The interaction between intrinsic PD-L1 expression and exogenous IFN-γ exposure enhances PD-1 therapy’s effectiveness in CRC by increasing CD8^+^ T-cell infiltration into the TME through modulation of the JAK-STAT pathway (Yuan et al., 2019).

Beyond pharmacological approaches, RT remains an effective nonsurgical option for patients with advanced CRC (Parikh et al., 2021; Hsieh et al., 2022; Shi et al., 2023). In a CRC xenograft mouse model, combining JAK2 inhibition with RT significantly reduces tumor growth. Targeting the JAK2-STAT3 pathway promotes apoptosis and reduces the clonogenic potential of RT-exposed CRC cells, such as HCT116 and LoVo, thereby improving therapeutic outcomes following radiotherapy (Park et al., 2019).

### 4.4 Challenges of targeting the JAK-STAT pathway in CRC

Despite extensive research into the mechanisms and therapeutic potential of targeting the JAK-STAT pathway in CRC carcinogenesis, no clinical drugs specifically aimed at this pathway have been developed for CRC treatment. A key challenge is the intricate crosstalk between the JAK-STAT pathway and other critical signaling cascades, such as PI3K-AKT and Wnt (Bose et al., 2020; Singh and Singh, 2020; Verhoeven et al., 2020). This interaction can activate compensatory mechanisms that undermine the effectiveness of JAK-STAT inhibitors, allowing tumor cells to circumvent therapeutic interventions. Furthermore, non-canonical JAK-STAT signaling represents a significant obstacle, as this alternative pathway can continue to promote CRC progression even in the presence of inhibitors designed to block canonical signaling, thus diminishing the overall treatment efficacy (Majoros et al., 2017; La Manna et al., 2021; Rah et al., 2022; Park et al., 2023). Additional challenges, such as issues related to drug bioavailability, tissue penetration, and specificity, further complicate the clinical application of JAK-STAT inhibitors (Bose et al., 2020; Favoino et al., 2021; Wang et al., 2024). Although numerous agents have shown promise in preclinical studies and animal models, their translation to clinical practice has been limited by concerns regarding definitive efficacy and the lack of sustained long-term benefits for patients (Yu et al., 2014; O’Shea et al., 2015; Trivedi and Starz-Gaiano, 2018; McLornan et al., 2021). To address these limitations, more comprehensive, large-scale clinical trials, alongside detailed animal studies, are essential to unravel the complexities of JAK-STAT signaling in CRC and to develop more effective therapeutic strategies.

## 5 Conclusion

The abnormal activation of the JAK-STAT pathway has been extensively documented in CRC and is closely linked to prognosis and various clinical features. A growing body of research underscores the pivotal role of JAK-STAT signaling in CRC progression, influencing critical processes such as tumor growth, metastasis, immune evasion, and the maintenance of cancer stem cell phenotypes. As the underlying mechanisms of JAK-STAT signaling in CRC are further elucidated, this pathway has emerged as a promising therapeutic target. Modulating the JAK-STAT pathway holds significant potential to enhance the efficacy of existing CRC treatments, including chemotherapy, immunotherapy, targeted therapies, and radiotherapy. However, several challenges continue to hinder the clinical translation of therapies targeting the JAK-STAT pathway in CRC. Issues such as bioavailability and pathway-specific selectivity complicate drug development. Addressing these limitations requires more rigorous research efforts, including multicenter clinical trials and comprehensive experiments, to better understand the molecular mechanisms driving JAK-STAT signaling in CRC and to evaluate the potential therapeutic benefits of targeting this pathway.
